# Crystallography on a chip – without the chip: sheet-on-sheet sandwich

**DOI:** 10.1107/S2059798318011634

**Published:** 2018-10-02

**Authors:** R. Bruce Doak, Gabriela Nass Kovacs, Alexander Gorel, Lutz Foucar, Thomas R. M. Barends, Marie Luise Grünbein, Mario Hilpert, Marco Kloos, Christopher M. Roome, Robert L. Shoeman, Miriam Stricker, Kensuke Tono, Daehyun You, Kiyoshi Ueda, Darren A. Sherrell, Robin L. Owen, Ilme Schlichting

**Affiliations:** a Department of Biomolecular Mechanisms, Max Planck Institute for Medical Research, Jahnstrasse 29, 69120 Heidelberg, Germany; b Japan Synchrotron Radiation Research Institute (JASRI), 1-1-1 Kouto, Sayo, Hyogo 679-5198, Japan; c RIKEN SPring-8 Center, 1-1-1 Kouto, Sayo, Hyogo 679-5148, Japan; d Institute of Multidisciplinary Research for Advanced Materials, Tohoku University, Sendai 980-8577, Japan; e Diamond Light Source, Harwell Science and Innovation Campus, Fermi Avenue, Didcot OX11 0DE, England

**Keywords:** serial crystallography, room-temperature data collection, low dose, fixed target, XFEL, Mylar sandwich chip, high throughput

## Abstract

Crystallography chips are fixed-target supports consisting of a film (for example Kapton) or wafer (for example silicon) that is processed using semiconductor-microfabrication techniques to yield an array of wells or through-holes in which single microcrystals can be lodged for raster-scan probing. Although relatively expensive to fabricate, chips offer an efficient means of high-throughput sample presentation for serial diffraction data collection at synchrotron or X-ray free-electron laser (XFEL) sources. Truly efficient loading of a chip (one microcrystal per well and no wastage during loading) is nonetheless challenging. The wells or holes must match the microcrystal size of interest, requiring that a large stock of chips be maintained. Raster scanning requires special mechanical drives to step the chip rapidly and with micrometre precision from well to well. Here, a ‘chip-less’ adaptation is described that essentially eliminates the challenges of loading and precision scanning, albeit with increased, yet still relatively frugal, sample usage. The device consists simply of two sheets of Mylar with the crystal solution sandwiched between them. This sheet-on-sheet (SOS) sandwich structure has been employed for serial femtosecond crystallography data collection with micrometre-sized crystals at an XFEL. The approach is also well suited to time-resolved pump–probe experiments, in particular for long time delays. The SOS sandwich enables measurements under XFEL beam conditions that would damage conventional chips, as documented here. The SOS sheets hermetically seal the sample, avoiding desiccation of the sample provided that the X-ray beam does not puncture the sheets. This is the case with a synchrotron beam but not with an XFEL beam. In the latter case, desiccation, setting radially outwards from each punched hole, sets lower limits on the speed and line spacing of the raster scan. It is shown that these constraints are easily accommodated.

## Abstract 2

Fixed targets or chips offer an efficient means of high-throughput microcrystal delivery for serial measurements at synchrotrons and XFELs. A low-background Mylar sandwich chip that alleviates the challenges of chip availability and crystal loading is described.

## 1. Introduction   

Serial femtosecond crystallography (SFX) at X-ray free-electron lasers (XFELs) is a new and unique way to collect diffraction data such that the limitations of radiation damage imposed by conventional X-ray sources are largely alleviated. SFX allows the acquisition of room-temperature data using small or weakly diffracting crystals, enabling time-resolved experiments, studies of membrane proteins and, in particular, studies of radiation-sensitive systems such as metalloproteins (Schlichting, 2015 ▸). XFELs provide femtosecond short pulses of very high peak brilliance that are used for data collection in a ‘diffraction-before-destruction’ approach (Neutze *et al.*, 2000 ▸; Chapman *et al.*, 2014 ▸). Since the microcrystals are destroyed upon a single XFEL exposure, many two-dimensional diffraction snapshots of data are acquired serially and are then merged into a three-dimensional data set. The rapid conveyance of fresh microcrystals into the sample–FEL interaction zone is imperative for efficient SFX data collection. Crystals can be delivered using a variety of methods that include liquid microjets from gas dynamic virtual nozzles (GDVNs; Weierstall *et al.*, 2012 ▸), medium-viscosity (Sierra *et al.*, 2016 ▸) to high-viscosity streams (Weierstall *et al.*, 2014 ▸; Botha *et al.*, 2015 ▸), droplets (Fuller *et al.*, 2017 ▸) or ‘chips’ (fixed targets; Zarrine-Afsar *et al.*, 2012 ▸; Mueller *et al.*, 2015 ▸; Roedig *et al.*, 2015 ▸; Baxter *et al.*, 2016 ▸; Hunter *et al.*, 2014 ▸). Particularly in view of the pulsed nature of XFEL sources, each of these approaches has its own advantages and disadvantages with regard to efficiency of sample use, magnitude of X-ray background, ease of performing time-resolved measurements, requisite expertise *etc.*

Recently, we set out to perform a time-resolved pump–probe experiment to follow microsecond-scale events in the allosteric R–T transition in haemoglobin (Hb) induced by ligand dissociation *via* flash photolysis of the light-sensitive haem iron–carbon monoxide (CO) bond. This approach is very similar to our previous ultrafast time-resolved work on CO dissociation from carbonmonoxymyoglobin (Barends *et al.*, 2015 ▸), where we used a liquid microjet for sample delivery. For very short time delays between the optical pump and XFEL probe pulses the microcrystals in the jet do not move appreciably, and the optical pump and X-ray probe can be overlaid spatially. At intermediate time delays Δ*t* the optical pump can be displaced by Δ*x* upstream towards the nozzle, such that photolyzed microcrystals reach the interaction zone at the desired time interval Δ*t* = Δ*x*/*v*, where *v* is the speed of the jet. For microsecond delays of up to about 5 µs, the optical pump beam can be positioned appropriately far upstream of the X-ray focus. Detailed knowledge of the jet speed (Grünbein *et al.*, 2018 ▸) is then critical to ensure that the probed crystals are properly probed. For still longer time delays, microcrystal delivery by GDVN liquid-microjet injection becomes impractical owing to the limited length of the contiguous liquid jet (prior to Rayleigh breakup) and the minimum jet speed required for stable jetting: the crystals are simply carried out of the XFEL interaction zone before the arrival of the next XFEL pulse. One solution to this problem is to slow the jet speed by using viscous additives or media (Botha *et al.*, 2015 ▸; Conrad *et al.*, 2015 ▸; Sugahara *et al.*, 2015 ▸, 2017 ▸; Kovácsová *et al.*, 2017 ▸). This was, however, not an option in our case since the very properties of the crystal form that allow Hb to undergo the full R–T transition also make the crystal packing very sensitive to any changes in the mother liquor. Since chip crystallography can simplify pump–probe time-resolved measurements by offering a stationary target as opposed to a moving target (crystals carried in a carrier stream which, particularly with high-viscosity carriers, can display significant fluctuations in speed), it was the preferred sample-delivery mode for SFX data collection for the Hb experiment.

‘Crystallography on a chip’ has attracted significant attention in recent years (Zarrine-Afsar *et al.*, 2012 ▸; Hunter *et al.*, 2014 ▸; Cohen *et al.*, 2014 ▸; Mueller *et al.*, 2015 ▸; Murray *et al.*, 2015 ▸; Roedig *et al.*, 2015 ▸, 2016 ▸; Oghbaey *et al.*, 2016 ▸; Baxter *et al.*, 2016 ▸; Owen *et al.*, 2017 ▸; Guo *et al.*, 2018 ▸). ‘Chip’ in this context refers to a micro-patterned support consisting of a film (for example Kapton) or a wafer (for example silicon) fabricated using semiconductor-processing techniques. Typically, the chip provides a regular array of microscopic cells (wells or through-holes) into which crystals can be loaded surrounded by their mother liquor. The chip can then be scanned across a tightly focused X-ray beam to record X-ray diffraction images. If a stepwise raster scan is employed, *i.e.* stepping from one array cell to the next, crystals need be present only in the cells. Sample usage is then reduced to an absolute minimum. A raster scan, either stepwise or continuous, can be synchronized to the pulse structure of an XFEL source to advance from one cell to the next between X-ray pulses. Provided that the crystals in the cells display sufficiently random orientations, the resulting SFX data collection can be highly efficient (Cohen *et al.*, 2014 ▸; Hunter *et al.*, 2014 ▸; Sherrell *et al.*, 2015 ▸; Mueller *et al.*, 2015 ▸).

Nonetheless, chips introduce their own set of unique challenges. Quite apart from the difficulty and expense of chip fabrication, loading crystals into the cells is often nontrivial. Wetting forces tend to repel crystals from wells or holes. In the case of through-holes, mild suction can be applied to the back of the chip as the crystal solution is spread on the front to pull the crystals gently into the holes (Oghbaey *et al.*, 2016 ▸). Trapping can be improved by tapering through-holes to form funnels from front to back (Mueller *et al.*, 2015 ▸) or by adding small through-holes in the well (Guo *et al.*, 2018 ▸). In lieu of suction, filter paper can be lightly pressed against the back of the chip to wick away the solvent (Guo *et al.*, 2018 ▸). Through-holes must obviously be matched in size to the crystals or the crystals will be pulled through the holes and lost. Size matching is further complicated by the minimum hole size, as set by the need for the X-ray beam (including any harmful wings) to pass through the holes without striking chip material. To avoid background scattering from the chip and/or damage to the chip itself, the raster scan across the chip must be exactly registered with the cell array, generally to micrometre accuracy over centimetres, which requires specialized mechanical drives. Independent of this, shock waves can be launched as the XFEL beam vaporizes the crystal solution within the holes. These shock waves can mechanically damage the chip, the neighbouring crystals or both [unpublished observations obtained at the Linac Coherent Light Source (LCLS)].

Chips clearly place stringent constraints on monodispersity of the crystal size, on the design and fabrication of the chip, and on the focal-spot size of the X-ray beam. To avoid these constraints, an alternative approach is to use a very thin substrate perforated by small holes that serve only as sieve holes to drain the liquid through the chip (*via* suction or blotting; Roedig *et al.*, 2016 ▸). Crystals are then deposited in a random fashion onto a planar substrate (generally silicon), which must be so thin as to introduce minimal X-ray scattering. Scanning then need not be made in registry with the holes. This considerable simplification comes at the cost of increased X-ray background and possibly even diffraction from the chip. Brittleness of the substrate can also become an issue. Regardless of whether the holes serve as crystal-collection points or only as drains, SFX requires that crystal retention in/on the chip presents sufficiently different crystal orientations to cover all of reciprocal space during data collection (Zarrine-Afsar *et al.*, 2012 ▸). When working with crystals in aqueous mother liquor, the loaded chip must either be cryocooled, tightly sealed or maintained in a suitably humid environment to prevent non-isomorphism or crystal damage by dehydration.

We fully intended to carry out a time-resolved pump–probe experiment on carboxyhemoglobin (Hb.CO) microcrystals using conventional crystallography chips. When this proved to be unachievable, for reasons that will become clear, we were forced to improvise. The result was the sheet-on-sheet (SOS) sandwich for crystal mounting, which we present here, describing SFX data collection from microcrystals of lysozyme (a well characterized model system) and haemoglobin. Although an operational response to a specific experimental impasse, it was also immediately clear that the SOS sandwich not only sidesteps most of the issues enumerated above but also eliminates microfabrication entirely. We characterized the performance of the SOS sandwich using SFX data collected at the SPring-8 Angstrom Compact free-electron LAser (SACLA) in Hyogo, Japan using 7.3 keV X-rays for SFX data collection and compared and contrasted its performance with that of the well established silicon chip (Mueller *et al.*, 2015 ▸; Owen *et al.*, 2017 ▸).

## 2. Experimental setup   

### 2.1. The silicon chip and the SOS sandwich   

Our conventional silicon chip had funnelled through-holes (Owen *et al.*, 2017 ▸), similar to that used previously for SFX data collection and described recently (Mueller *et al.*, 2015 ▸; Oghbaey *et al.*, 2016 ▸) but with an updated layout and a new fabrication process. The ∼30 × 30 mm chip was mounted between two 2.5 µm thick sheets of Mylar polyester pressed together by two O-rings at the periphery of the frame to hermetically enclose the volume within the sheets and so seal the chip and sample to avoid dehydration. The Mylar sheets were in direct contact with the chip faces.

The SOS sandwich consisted simply of two thin polymer sheets with a thin layer of sample solution sandwiched directly between them. Mylar sheets of 2.5 µm thickness were employed, mounted in an identical chip holder to that of the silicon chip (Owen *et al.*, 2017 ▸), simply without the chip. The Mylar sheets were again pressed tightly together by the two O-rings around their periphery to hermetically seal the sample between the sheets. A dedicated holder and loading jigs for SOS sandwich use were subsequently designed and fabricated (Supporting Information §S3).

Mylar is very resistant and impermeable to most gases and liquids, and absorbs only negligible amounts of water on time scales of many hours (Axford *et al.*, 2016 ▸). Once sealed within Mylar, the sample is therefore expected to remain hydrated for several hours. During XFEL measurements, each X-ray pulse burns a hole through the Mylar sheets, breaching this seal. In the case of mother liquors containing high concentrations of salt, the resulting dehydration may be visible in real-time camera monitoring as a spreading circular region around each XFEL strike point (Supporting Information §S2). It is imperative that the chip raster scan ‘outruns’ the spread of all such dehydration zones, such that no XFEL pulse samples a region dehydrated by a previous XFEL pulse.

### 2.2. Sample loading of the chip and sandwich   

Human oxyhaemoglobin A (Hb.O_2_) was purified from expired units of human blood (type A) as described previously (Antonini & Brunori, 1971 ▸; Perutz, 1968 ▸) and converted to the carbonmonoxy complex as follows. A three-necked flask was equipped with a magnetic stirring bar, two gas inlets with stopcocks and a rubber stopper, and was charged with the HbO_2_ solution. Upon repeated cycles of evacuation (5–10 min) and flushing with CO using a Schlenk line, the tomato-red protein solution turned raspberry red. No sodium dithionite was added. Rod-shaped Hb.CO microcrystals grew in a CO-saturated atmosphere at room temperature within a few days upon mixing solutions of Hb.CO (∼2 m*M* in water) and precipitant (3.2 *M* NaH_2_PO_4_ and 3.2 *M* K_2_HPO_4_ in a 2:1 ratio) in a ratio of 1:2.5. No toluene was added. Lysozyme microcrystals were prepared as described previously (Barends *et al.*, 2014 ▸) except that the crystals grew at 20°C, resulting in larger crystals (3 × 3 × 3 µm). In brief, 2.5 ml protein solution [32 mg ml^−1^ hen egg-white lysozyme (Sigma) in 0.1 *M* sodium acetate buffer pH 3.0] and 7.5 ml precipitant solution (20% NaCl, 6% PEG 6000, 0.1 *M* sodium acetate pH 3.0) were mixed rapidly and left overnight at room temperature on a slowly rotating wheel shaker. After gravity-induced settling, the crystalline pellet was washed several times in crystal-storage solution (10% NaCl, 0.1 *M* sodium acetate buffer pH 4.0).

**Figure 1:**
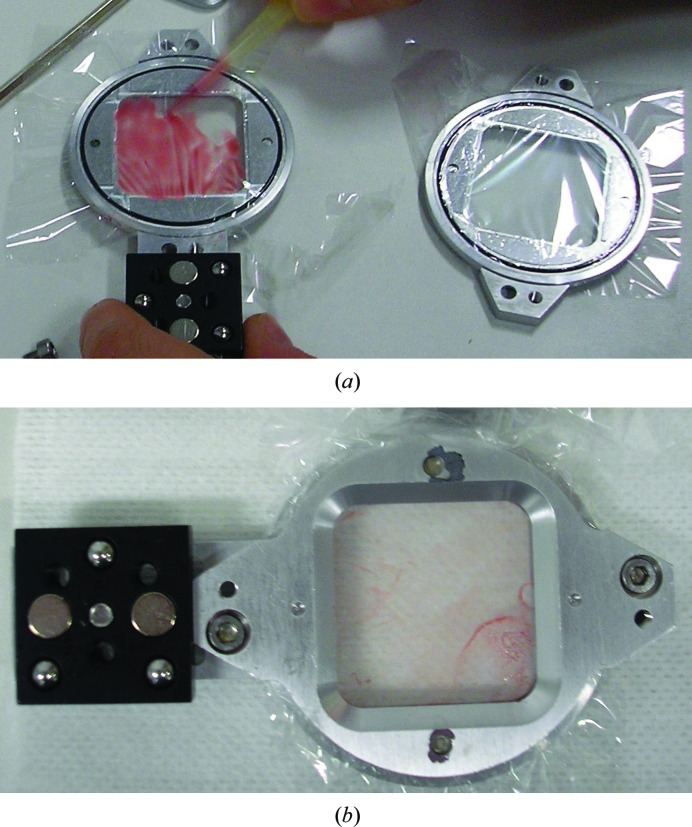
(*a*) Loading the SOS sandwich based on the chip holder (Owen *et al.*, 2017 ▸); (*b*) loaded SOS sandwich. A larger than necessary 25 µl sample volume is shown being loaded in these images.

A sheet of Mylar was stretched across each of the two half-frames of the chip holder (Owen *et al.*, 2017 ▸), gently pulled taut to eliminate wrinkles and clamped in place with alu­minium clamping rings. A 14 µl volume of microcrystalline pellet (1/4 settled material) was pipetted onto the silicon chip or, in the SOS case, directly onto the lower Mylar membrane (2.5 µm thickness) and was then spread to a thin layer with a pipette tip (Fig. 1 ▸). The two frame halves were then placed into contact, leading to further spreading and thinning of the film by capillary action, and the frame was sealed together with four clamping screws. Initial sample loading was performed in a tent with 70% humidity, which was achieved using a humidifier. Since no drying of the sample was observed in the short time needed to load the chip, and no difference was observed in the diffraction data when loading was performed outside the tent, the latter approach was used for the majority of the beamtime.

### 2.3. Data collection and analysis   

The experiment (proposal No. 2017B8002) was performed in December 2017 at SACLA in Hyogo. SFX data collection was performed on beamline 2 (BL2) in the large helium chamber using a multiport charge-coupled device (MPCCD) detector (Kameshima *et al.*, 2014 ▸). SACLA operated at 30 Hz and delivered X-ray pulses of 10 fs duration and a nominal photon energy of 7.3 keV (λ = 1.770 Å) with 0.48–0.50 mJ average pulse energy. The beamline transmission was 70%.^1^ No attenuators were used. The focal spot size was measured to be 1.4 µm (vertical) × 1.6 µm (horizontal) FWHM. The Diamond Light Source chip holder (Owen *et al.*, 2017 ▸) was used for both the silicon chip and the SOS sandwich. The Diamond mini-endstation was used for data collection (Sherrell *et al.*, 2015 ▸). Online data analysis and offline hitfinding was performed with *CASS* (Foucar, 2016 ▸). The detector metrology was optimized in two steps. Firstly, the detector panel alignment was optimized (see Barends *et al.*, 2015 ▸). The detector–XFEL interaction zone distance was optimized by a parameter grid search minimizing the root-mean-square deviation between reflections and diffraction peaks as measured by the *geoptimiser* tool (Yefanov *et al.*, 2015 ▸) from the *CrystFEL* software suite (White *et al.*, 2012 ▸). *CrystFEL* v.0.6.3 (White *et al.*, 2012 ▸) was used for further offline data analysis. The data were phased by molecular replacement with *Phaser* (McCoy *et al.*, 2007 ▸) using PDB entry 4et8 (Boutet *et al.*, 2012 ▸) and PDB entry 1mko (Safo & Abraham, 2005 ▸) as the search models for lysozyme and Hb.CO, respectively, and were refined using alternating cycles of rebuilding in *Coot* (Emsley *et al.*, 2010 ▸) and refinement in *REFMAC*5 (Murshudov *et al.*, 2011 ▸).

## 3. Results and discussion   

**Figure 2:**
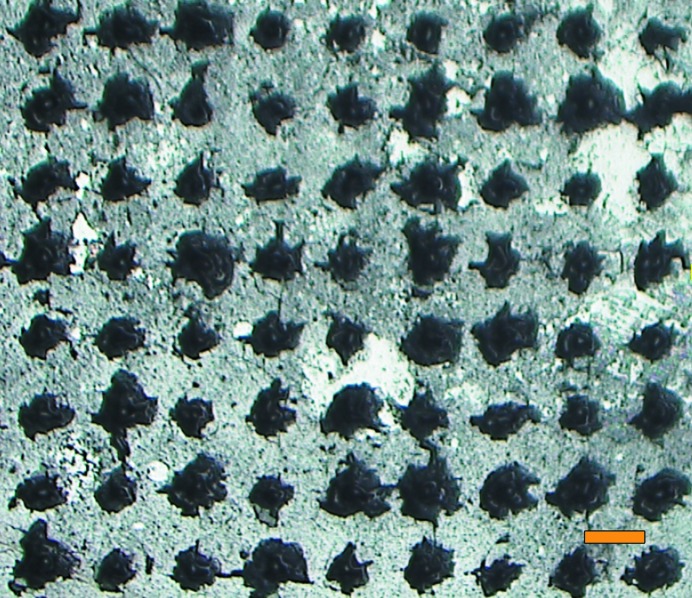
A silicon chip exposed to an unattenuated XFEL beam at 7.3 keV photon energy at SACLA. The beam (1.4 × 1.6 µm FWHM) was centred in the wells (7 × 7 µm). Nevertheless, significant damage to the chip was observed. The chip is shown from the back after exposure. The scattered silicon powder resulted in significant diffraction. In some chips, the accumulated stress was so large that the chip fractured. The orange scale bar corresponds to 100 µm.

Chip development to date has concentrated largely on silicon and/or silicon nitride, given their compatibility with semiconductor-processing techniques. However, these supports can contribute both background and diffraction to the measured diffraction images. This can be minimized by using very thin silicon films (Roedig *et al.*, 2015 ▸), which are however fragile and shatter easily. An amorphous material of low atomic number and low density is preferable. Indeed, polyimide (commercialized as Kapton) supports are available commercially (MiTeGen) in various forms and shapes and are used as micromounts. Recently, the well established micromesh mount was extended to a micro-sized well mount (Guo *et al.*, 2018 ▸). Its small size (diameter 250 µm) is advantageous for cryocooling and rotation data collection at synchrotron sources but is a disadvantage for room-temperature SFX data collection at XFELs. This motivated us to instead choose a silicon chip that has been used before for serial data collection at XFELs (Mueller *et al.*, 2015 ▸; Oghbaey *et al.*, 2016 ▸) and synchrotron sources (Owen *et al.*, 2017 ▸). Given the high optical density of Hb crystals (protein concentration 50 m*M*), small crystals (∼7 ± 3 µm) are required for a reasonable photolysis yield. Since our Hb microcrystals diffracted only weakly and the detector quantum yield of the MPCCD detector available during our beamtime depends strongly on photon energy, we chose to use 7.3 keV photon energy without attenuators. The Hb.CO microcrystals were loaded into a silicon chip with 7 µm well size and sealed with 2.5 µm Mylar sheets as outlined above. The chip was carefully aligned to the X-ray beam using optical fiducials as recently described (Owen *et al.*, 2017 ▸) to ensure accurate alignment of the raster scan with the chip hole array. Nonetheless, we observed strong degradation of the chip as the raster scan proceeded. Post-scan microscope imaging showed the XFEL scan to have indeed been aligned with the chip holes, yet the holes had been severely enlarged and damaged (see Fig. 2 ▸ and Supporting Information §S1). Debris from this interaction was even deposited onto the Mylar foil over wells that had not yet been exposed, resulting in a silicon powder background when these positions were subsequently probed. Since the X-ray beam (1.4 × 1.6 µm FWHM) was nominally much smaller than the 7 µm diameter chip holes, the unavoidable conclusion was that under the experimental parameters required for the experiment (7.3 keV photon energy, unattenuated beam) tails of the X-ray focus beyond the FWHM sufficed to damage the chip. It is most likely that this damage is related to the X-ray photon energy of 7.3 keV and the fact that we used the unattenuated beam. At 7.3 keV ∼65% of the photons are absorbed compared with ∼30% at 10 keV. Indeed, previous experiments using the silicon chip performed at 10 keV with a 13% transmission beam did not show this damage (Supporting Information §S1; Supplementary Fig. S1). However, since our small weakly diffracting crystals required almost the full flux at 7.3 keV to yield good signal-to-noise data, another solution for data collection had to be found.

**Figure 3:**
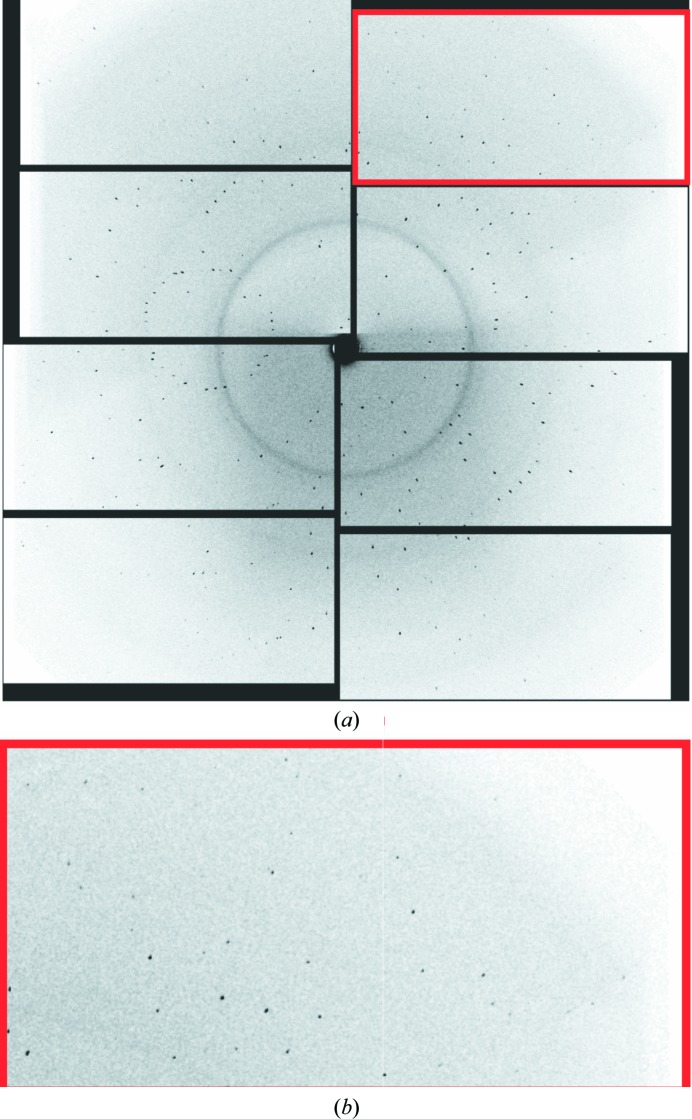
(*a*) Typical diffraction image for lysozyme microcrystals, 2–3 µm in size, mounted in the SOS sandwich. The corner of the detector corresponds to 2.0 Å resolution, with strong diffraction peaks (*b*). The diffuse scattering ring (4.7 Å) originates from the two 2.5 µm thick Mylar sheets. XFEL beam at 7.3 keV photon energy, 480–500 µJ pulse energy, 70% beamline transmission, 10 fs duration, (1.4 × 1.6 µm FWHM) XFEL spot, 30 Hz pulse repetition rate.

**Figure 4:**
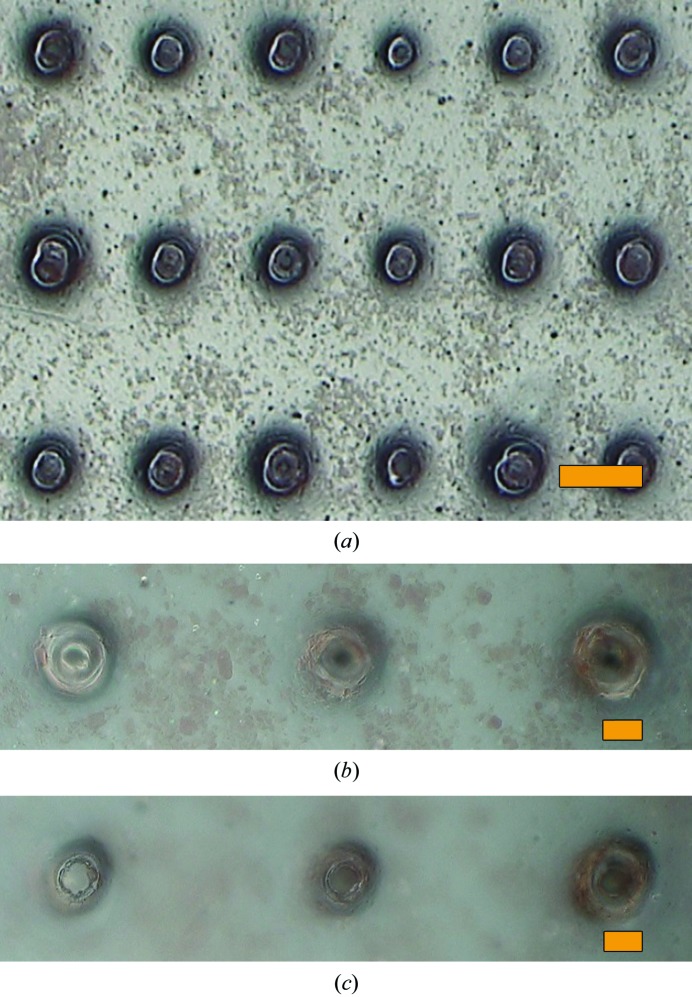
(*a*) Microscope image of the SOS sandwich loaded with haemoglobin microcrystals (brown granular material) after exposure to unattenuated XFEL pulses at 7.3 keV photon energy. (*b*, *c*) Enlarged views focused on the crystal layer (*b*) and on the edges of through-hole burned by the XFEL (*c*). Here, the stepwise raster scan was 125 µm on centres horizontally and 250 µm vertically. The orange scale bar corresponds to 100 µm in (*a*) and 20 µm in (*b*) and (*c*).

This impasse suggested foregoing the chip entirely and simply sandwiching the microcrystal-containing liquid between two thin sheets of Mylar: a ‘chipless’ chip. For this purpose we employed the same 2.5 µm thick Mylar sheets employed to seal the chip along with the same holders. The primary points of concern were (i) whether the sample solution could be spread between the two sheets as a uniform thin film of no more than a few micrometres in thickness; (ii) whether dehydration of the sample could be avoided, both up until and during the XFEL scan; (iii) whether the presence of Mylar sheets in direct contact with the crystal solution would adversely affect the crystals; and (iv) how well the Mylar sheets would stand up to the XFEL pulses. We explored these concerns using lysozyme microcrystals, a very well established model system for SFX measurements (Boutet *et al.*, 2012 ▸; Barends *et al.*, 2014 ▸; Gorel *et al.*, 2017 ▸). The results proved to be quite encouraging, prompting us to investigate Hb.CO microcrystals using the same sheet-on-sheet (SOS) mounting method. The mother liquors of the haemoglobin and lysozyme microcrystals contained high concentrations of salt (1.6 *M* phosphate for haemoglobin and 1.7 *M* NaCl for lysozyme). Nonetheless, both systems could easily be ‘loaded’ as a thin layer between SOS sheets and both samples delivered well defined diffraction images, with no indication of damage from the loading process. The Mylar sheets give rise to an easily discernible narrow ring at 4.7 Å resolution, but of sufficiently low intensity as to cause no difficulties in data analysis. A resulting diffraction pattern for lysozyme is shown in Fig. 3 ▸. The hit rate typically varied from 10 to 30%. The unattenuated XFEL X-ray pulses burned microscopic holes through the films, typically of inner diameter 15–20 µm and with a ‘crater’ region surrounding this out to a diameter of roughly 40–50 µm (Fig. 4 ▸). The through-hole and crater radii were larger in the regions where the XFEL beam encountered larger crystals or a thicker layer of sample solution, evidently owing to the increased X-ray absorption at such positions. Dehydration by solvent evaporation through the XFEL burn-holes proceeded fairly slowly, remaining spatially confined to near the holes on time scales of minutes. This was despite the fact that the hutch temperature was 30°C and that the mother liquor contained ∼1.7 *M* salt. Lysozyme crystals are highly sensitive towards dehydration, resulting in strong non-isomorphism (Takayama & Nakasako, 2011 ▸). Nevertheless, we did not observe any indications of non-isomorphism in the data collected using the SOS sandwich.

The data-collection statistics for one lysozyme chip are listed in Table 1 ▸. This run took just over 14 min (the time needed to fully scan one loaded SOS assembly at a 30 Hz XFEL repetition rate) and yielded 14 000 indexed images, *i.e.* ∼1000 images per minute. Since a volume of 14 µl of sample had been loaded onto the chip, the sample usage was 1.0 nl per indexed image. We did not explore the use of a smaller sample volume. This should be possible when working in a controlled humid atmosphere. We did not detect any influence of the raster spacing (horizontal step size 30, 50, 75 and 100 µm; vertical step size 125 and 250 µm) on data quality. The lysozyme data listed in Table 1 ▸ were collected using a raster scan spacing of 50 µm on centres along horizontal rows and 125 µm on centres between rows, which was the densest row spacing employed in these SOS measurements, and therefore that most likely to show any damage effects. The Hb.CO data (Table 1 ▸) were collected using a raster-scan spacing of 140 µm on centres along horizontal rows and 250 µm on centres between rows. The wide spacing was chosen to prevent accidental pre-illumination when collecting optical pump–X-ray probe data (to be reported elsewhere).

High-efficiency chips (those intended to be loaded with one microcrystal per well/hole) are generally deemed to be advantageous when the availability of sample is the limiting experimental factor. Quantification of this efficiency is difficult to assess. We have been unable to find any information on the fraction of crystals lost in the suction/blotting process or the fraction that deposit on the chip but fail to lodge in the wells/holes. One would expect, at least for polydisperse collections of small microcrystals, that much of the applied sample could easily be lost to one of these factors. If through-holes are poorly matched to crystal size, chips that employ holes only as drains and not as localization sites will generally be less efficient than SOS by the fraction of sample lost in the drain process. SOS imposes no intrinsic lower limit on crystal size. For chips employing through-holes as crystal localization sites the holes must be significantly larger than the X-ray focal spot. This sets a rigorous lower limit on crystal size. Our rough model calculations suggest that conventional chips with crystals located in through-holes are about two orders more efficient in sample usage than the SOS sandwich, which itself is predicted to be more efficient than GDVN by about two orders of magnitude. High-viscosity extrusion (HVE) injection should be almost as efficient as chips. Interestingly, GDVN at a continuous megahertz repetition rate (if this proves to be feasible) is predicted to rival chips and HVE in the efficiency of sample usage, but at data collection rates that are three orders of magnitude faster.

The specific merit of SOS is therefore clear. Particularly if the availability of sample is not the overriding concern, SOS allows the experimental complexity and cost to be reduced significantly. Modest mechanical drives suffice for the raster scan, since this need not be made in exact registry with an array of micrometre-sized wells/holes. ‘On-the-fly’ scans are possible for the same reason, allowing much faster scanning than with well-to-well stepping. Certain chip-imposed restrictions on experimental parameters (XFEL wavelength, power and focus, crystal size and distribution *etc.*) are elimin­ated. Room-temperature measurements are very easily carried out, with no external humidification required. The Mylar sheets deliver entirely tractable background scattering (Figs. 3 ▸ and 5 ▸), while other polymer materials such as polypropylene or Parylene C deliver even less. As additional experience is accumulated, the technique is likely to prove amenable to a wide variety of crystal mother liquors/storage solutions over a wide range of viscosities and hydrophobicities, and for crystals of different types, sizes and shapes. This includes membrane-protein crystals grown in lipidic cubic phase (LCP). By use of a simple mechanical press, this highly sticky and viscous mesophase can be spread thinly between the SOS sheets without damaging either the crystals or the sheets (Supplementary Fig. S5). The SOS sandwich thus provides a very attractive and easily accessible alternative to HVE injection. Indeed, it even offers certain advantages by alleviating or bypassing specific problematic HVE injection behaviours such as nozzle clogging and irregular jet speed. This is especially advantageous for time-resolved measurements.

The use of a flexible polymer film rather than a brittle semiconductor sheet, as demonstrated previously (Hunter *et al.*, 2014 ▸; Coquelle *et al.*, 2015 ▸), greatly simplifies the mounting and sealing of sample, allows sample visualization before, during and after XFEL data collection, is less likely to damage fragile crystals and can markedly improve resistance to XFEL-induced damage. The microcrystals never leave their mother liquor during loading, which is so rapid and easy that a humidity-controlled environment appears to be unnecessary. The loading itself is much simpler than with conventional crystallography chips and does not appear to introduce any sample damage. There is little or no movement of the crystals once loaded. Sealing to prevent dehydration is integral to the loading, rather than a separate step (or series of steps), and requires no additional apparatus. The background owing to the surrounding layer of mother liquor is entirely tractable; it is possibly not as low as for chips with drain holes, but the latter are more difficult to mount, may lead to dehydration and impose constraints on crystal size.

As with chip crystallography in general, the SOS sandwich is highly suited to laser pump–probe time-resolved measurements, limited only by scan parameters as dictated by damage and spreading of desiccation. Even at the maximum 120 Hz XFEL pulse rate of first-generation XFEL sources, delays of many milliseconds are possible. Although not as frugal with sample as high-efficiency chips, SOS sandwiches are nevertheless relatively thrifty and this is likely to improve as ‘on-the-fly’ scanning is implemented. Since the size of the SOS sandwich can be scaled easily, it is equally as useful for serial rotation data collection at synchrotron sources as for SFX at XFELs.

In short, a sober assessment of chip crystallography suggests that its liabilities outnumber its advantages in all but those cases in which sample availability is an overwhelming experimental concern. We anticipate that SOS sandwiches will rapidly come to dominate a large portion of fixed-target measurements.

## Supplementary Material

Supporting text and Supplementary Figures.. DOI: 10.1107/S2059798318011634/tz5098sup1.pdf

## Figures and Tables

**Figure 5:**
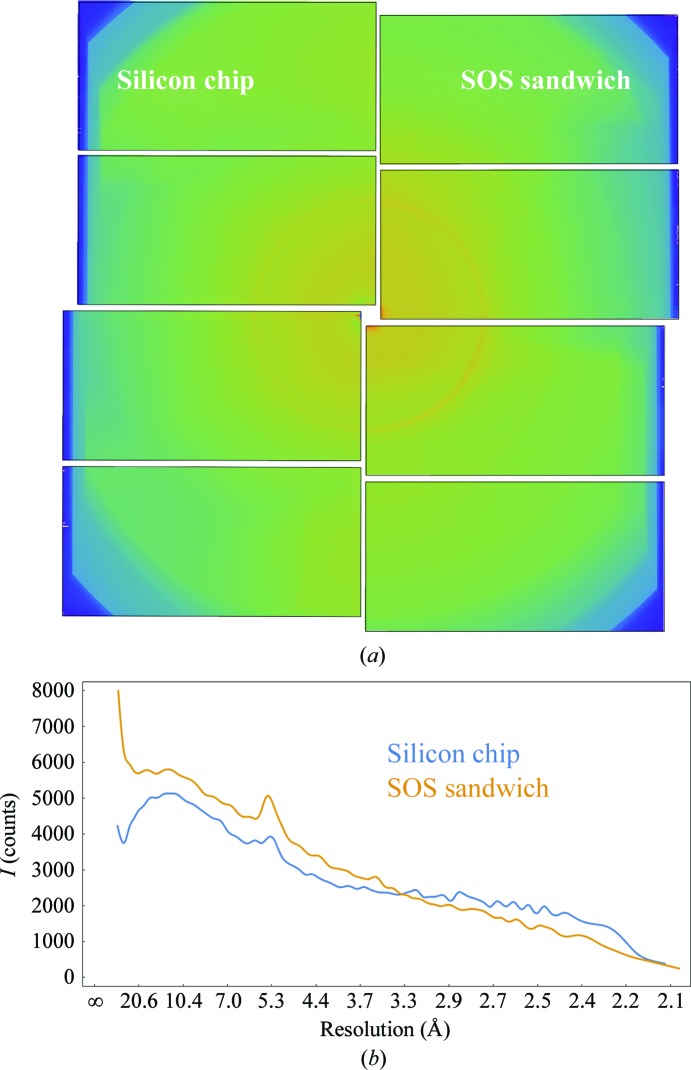
(*a*) Background of the silicon chip and SOS sandwich. The signal was calculated as the median of 1000 diffraction images (hits) of haemoglobin microcrystals mounted in the respective device for SFX data collection. The plot of the radial distribution is shown in (*b*).

**Table 1:** SFX data-collection and refinement statistics for data collected using the SOS sandwich Values in parentheses are for the highest resolution shell.

	Lysozyme	Hb.CO
Data-collection statistics		
Space group	*P*4_3_2_1_2	*P*2_1_2_1_2_1_
*a*, *b*, *c* (Å)	79.8, 79.8, 38.7	55.8, 157.4, 64.2
α, β, γ (°)	90, 90, 90	90, 90, 90
No. of crystals	13802 [73% indexing rate]	26039 [38% indexing rate]
Resolution range (Å)	40–2.1 (2.20–2.10)	39.3–2.2 (2.28–2.20)
Completeness (%)	100 (100)	99.96 (100)
Multiplicity	283 (42)	319 (65)
〈*I*/σ(*I*)〉	10.3 (2.8)	7.3 (2.2)
*R*_split_ †	0.090 (0.324)	0.115 (0.466)
CC	0.986 (0.773)	0.980 (0.654)
CC*	0.996 (0.934)	0.995 (0.889)
Wilson *B* factor (Å^2^)	33.3	42.3
Refinement statistics		
*R*/*R*_free_	0.199/0.235	0.182/0.212
R.m.s.d.		
Bond lengths (Å)	0.006	0.009
Bond angles (°)	0.962	0.980
No. of atoms		
Protein	992	4332
Water	44	20
Average *B* factors (Å^2^)		
Protein	31.2	43.5
Water	36.3	36.6
Percentage of residues in areas of Ramachandran plot		
Preferred	96.0	98.9
Allowed	4.0	1.1
Disallowed	0.0	0.0
PDB code	6gf0	6hal

† *R*_split_ = 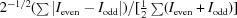 (White *et al.*, 2012 ▸), where *I*_even_ and *I*_odd_ are intensities determined from all even-numbered and all odd-numbered images, respectively.

## Footnotes

1 During the beam time the beamline had four silicon mirrors (2 mrad angle), four diamond films for beam monitoring (each ∼15 µm thick) and four beryllium windows (each ∼60 µm thick). The distance from the exit port of beamline to the sample was ∼0.4 m, where the atmosphere was ∼90% He and ∼10% air. Altogether, this resulted in a beamline transmission of 70%.
