# Supplementary material for: Crystallography on a chip – without the chip: sheet-on-sheet sandwich
Source: Acta Crystallogr D Struct Biol. 2018 Oct 2;74(Pt 10):1000–7. doi: 10.1107/S2059798318011634 (PMC6173051; doi:10.1107/S2059798318011634)
Supplement: Supplementary file 1 [file d-74-01000-sup1.pdf]

STRUCTURAL
BIOLOGY

Volume 74 (2018)

Supporting information for article:

Crystallography on a chip – without the chip: sheet-on-sheet sandwich

R. Bruce Doak, Gabriela Nass Kovacs, Alexander Gorel, Lutz Foucar, Thomas R. M. Barends, Marie Luise Grünbein, Mario Hilpert, Marco Kloos, Christopher M. Roome, Robert L. Shoeman, Miriam Stricker, Kensuke Tono, Daehyun You, Kiyoshi Ueda, Darren A. Sherrell, Robin L. Owen and Ilme Schlichting

S1. Damage to conventional crystallography chips during SACLA beamtime

Under the X-ray parameters required for our experiment (10 fs pulse duration, 7.3 keV photon energy, 480-500 μJ pulse energy, 100% transmission), the SACLA XFEL beam severely damaged the silicon support layer of chips of the Diamond group. To investigate whether this damage was related to absorption by silicon, we also tested a chip made of an organic polymer, Cyclotene, fabricated by the Semiconductor Laboratory of the Max Planck Society (details to be published). Damage to the silicon chip is documented in Figure S1 showing images taken before (a) and after (b) exposure to the XFEL beam at 7.3 keV, 480-500 μJ average pulse energy (70 % beamline transmission, no further attenuation). The chip on the top had actually been used at SACLA in an earlier beamtime, with no damage to the chip at that time in repeated exposures to the SACLA XFEL beam at a transmission of 13% at 10 keV. Exposure without attenuation at 7.3 keV damaged it as shown in Figure S 1(b), with the original 7 μm diameter through-holes of the chip expanded to 40 μm diameter or larger. Significant silicon diffraction peaks appeared in the recorded diffraction images and remnants of molten silicon were even seen in some of these holes. After this damage was observed, it was carefully verified that the XFEL focal spot was indeed the expected 1.5 μm "top-hat" diameter and that the scan was in exact registry with the chip holes. Accordingly it appears that the tails of the XFEL spot profile sufficed to initiate this damage. When the X-ray transmission was reduced to 18.6% as a check, the damage was less but not negligible.

Damage to the Cyclotene chips is illustrated in Figure S2. To fabricate these chips, a layer of Cyclotene polymer is spun onto a silicon wafer to a thickness of 5 micron and then cross-linked, after which the silicon is etched completely through to the Cyclotene-silicon interface to leave only the Cyclotene as the support layer for sample solution. The Cyclotene film is glassy in structure and so, unlike silicon, cannot contribute Bragg diffraction to the X-ray background. The fabrication is designed to leave the Cyclotene in a neutral state of tension/compression to mitigate formation of tears when exposed to the X-ray beam. The film may end up slightly buckled, but by no more than ~1 μm amplitude. The Cyclotene film is fairly robust. When used at synchrotrons, the chips can generally be cleaned and reused several times before the windows break. Drain holes are etched through the Cyclotene film to allow through-blotting during sample loading. No explicit attempt is made to capture and localize crystals in these through-holes or to scan across the chip in registry with the hole. The silicon serves only as a framework to support the polymer film. The chip is placed in a snap-together holder that is sealed front and back with 2.5 μm thick Mylar® films, through which the XFEL beam passes. The Mylar® films stand 1 and 4 mm away from the Cyclotene on the detector and X-ray source sides, respectively.

The Cyclotene chip shown in Figure S2 incorporates six Cyclotene windows, each with an array of 156 x 108 blotting holes spaced 75 μm on centres (array area 5.925 x 4.925 mm), giving 16,848 through

holes per window. The hole array consistes of two different alternating sizes, 10 μm and 20 μm square on this chip. The windows of Figure S2 were probed with the XFEL in the order A through F as labelled in the figure, stepping the chip 200 μm along a horizontal line, dropping 200 μm vertically, and then stepping back along the next horizontal row in the reverse direction. Good diffraction images were obtained from window A of this chip but the diffraction quality then deteriorated progressively and substantially through the remaining windows. In part this seemed to be due to volatilized sample solution re-depositing on the support film and Mylar® sealing films further along the chip from the window being scanned. Holes burned through the back Mylar® film were significantly larger in regions where this build-up was evident. Windows B, C, E, and F ruptured during the XFEL exposure, although apparently fairly late in each window scan, since arrays of XFEL burn holes could be seen even in the torn portions of the Cyclotene films. An enlarged view of the holes punched by the XFEL through window A of Figure S2A is shown in Figure S3. The holes are 5-15 μm in diameter, with spider cracks radiating radially outwards from the hole edges as far as 50-100 μm. The film ruptures presumably once cracks from adjacent shots meet. The “bleached“ regions surrounding each XFEL punch hole are likely not due to the XFEL beam but rather to the optical pump laser used in these experiments.

S2. Damage propagation: Implications for raster scanning

The energy deposited by an XFEL pulse varies with wavelength and energy of the beam and with the type and thickness of the material being irradiated. In general, even for very thin films, sufficient energy is deposited to generate a fully ionized plasma that expands explosively into the surrounding medium. Damage can be transmitted mechanically (pressure/shock waves), by ballistic transport of energetic species (neutrals, ions, or electrons), or by slow diffusion of radicals excited by plasma. Each such form of damage propagates radially outward at some characteristic speed. Other processes, such as dehydration of the sample via evaporation through the holes punched by the XFEL pulses, behave similarly. Since a raster scan necessarily involves periodically approaching previously irradiated positions, the scan parameters must be chosen such that no XFEL pulse samples a damage zone by a previous pulse. In the scans employed in this work, the chip is rastered along a horizontal line scan, e.g., from left to right, stepped downwards by a chosen distance, rastered right to left, stepped downwards, etc. The critical time for damage propagation is then that from the beginning of one horizontal transit to the ending of the subsequent. The downwards step between horizontal line scans must exceed the distance travelled by all damage mechanisms in this time period. An instructive example is provided in Figure S4 for circular desiccation zones around the holes burned by the XFEL beam through the films. The ongoing horizontal line scan is at the bottom of this image, with the scans higher on the image from bottom to top having been made progressively earlier. Desiccation of this lysozyme sample gave rise to easily discerned discoloration, observed as a circular zone around each

XFEL strike point. These disks are progressively larger from bottom to top of the image, reflecting the spread of the desiccation zones with time, and it is clear that the chosen line separation easily sufficed to avoid sampling desiccated zones.

S3. SOS frame and SOS loading jig

Although the Diamond chip holder worked extremely well as an SOS frame for these measurements, a specialized SOS holder was subsequently designed. Drawings for this mounting framework and its matched loading jig are provided in Figures S6 and S7. The mounting frame consists essentially of two 50 mm square open frames that compress the SOS sheets against one another and against an O-ring to hermetically seal the region between the sheets. Small disk magnets embedded in the frames provide a weak clamping force that holds the two plates together lightly during after loading, yet allows the sheets to be pulled taut around the periphery to remove wrinkles. Eight screws, inserted as the last step in loading, clamp the sheets strongly together and provide effective sealing. Tightening the screws compresses the O-ring, pulling the sheets downwards very slightly around the periphery of a slight pedestal on the base plate. This leaves the sheets under slight tension and therefore planar. A tab on the bottom plate is provided for mounting the chip. Alternatively the chip can be mounted magnetically via the frame magnets. The frame has minimal mass and so is compatible with high accelerations for rapid back-and-forth raster scans.

To load the SOS sandwich, the back frame, with its magnets and O-ring in place, is inserted into a matching recess in a loading plate. The back SOS film is then laid gently over the recessed plate and a narrow disk cut from flexible ferrite magnetic sheet is laid over the sheet, to give a light clamping force by attraction to disk magnets embedded in the loading plate. This holds the film in place and allows its surface to be pulled taut to remove any wrinkles. A raised pedestal in the centre of the recess is in direct contact with film to allow pipetting and spreading of sample onto the sheet without danger of puncturing the sheet. The second sheet is then laid carefully onto the first. As when placing a cover slip onto sample on a microscope slide, the sample applied to the back sheet immediately spreads via capillary action into a very thin film between the sheets. If necessary, the upper film is tugged taut to remove any wrinkles and the front frame then placed onto the stack, aligned by a rim around the central hole of the back sheet. The embedded magnets hold the two frames together as the eight screws are inserted and tightened. Using a scalpel, the sheets are then trimmed around the periphery of the frame and the SOS is ready to use. The entire loading process takes only a few minutes. Once loaded and sealed, the chips appear to maintain solvation for several hours. Prototypes of the mounting frames were 3D-printed in polylactic acid (PLA) to test the design, the loading procedure, and the efficacy of sealing. The PLA version appeared to be of sufficiently high quality for actual experimental use.

S4. Mounting high viscosity samples in the SOS sandwich

Using membrane microcrystals in LCP, we tested the loading of high viscosity sample solutions into the SOS sandwich (Figure S5). The loading procedure necessarily differs from that of low viscosity liquids, since high viscosity media is difficult to spread mechanically onto an SOS sheet and furthermore does not spread under capillary action when sandwiched between two sheets. Some flattening of a loaded sample is possible by gently squeezing the SOS sheets between thumb and forefinger. Fairly thin (60-120 μm) layers can be obtained in this manner, but non-uniformity of thickness is a problem and the danger of tearing the Mylar sheets is high. We therefore assembled a rudimentary mechanical press, (see Figure S5b), for thinning a viscous layer once sandwiched between SOS sheets. Since this compression requires surfaces that are flat and smooth to micrometre resolution, microscope slides were used as the thrust and base plates of the press. The former was a 25.7 mm square and 1 mm thick glass plate, cut from a standard microscope slide and bevelled on the corners to avoid damaging the SOS films. The latter was simply a 22 mm sq. and 0.17 mm thick cover slip, laid atop the SOS loading jig. The thrust plate was attached to a 25 mm drive shaft with 1/16 in thick double-sided tape, which accommodated any slight misalignment of the two plates. A hand-driven translation stage drove the two plates together with a force of up to about 20 N. To allow trapped gases to escape, the sheets were left unsealed around the periphery while the sample layer was being compressed. The upper SOS frame was then screwed into place to seal the assembly. Tightly-spaced Newton’s rings were observed around the periphery of the sample, indicating that a few micrometres of air were trapped between the sheets in that region. A more sophisticated press design could likely eliminate this, either by simply increasing the plate sizes or by rolling rather than pressing. Although we have not yet attempted to do so, it is possible that *low* viscosity samples could also be spread and thinned in this manner, thereby reducing X-ray scattering background.

A compressed layer of bacteriorhodopsin microcrystals in LCP (prepared as described in (Kovacsova *et al.*, 2017) is shown in Figure S5 (c, d). A deposited 20 μl volume over-fills the press plates when compressed, extruding beyond the plates to produce the thicker rim around the compressed sample region. Immediately after compression, the layer thickness was measured at several points using a high power microscope and found to be 40-60 μm. If spread uniformly to a thickness of 50 μm, a 20 μl volume would yield a disk of 23 mm diameter, in reasonable agreement with our measurement. Careful microscopic characterization of the loaded and compressed SOS sandwich showed no indication of mechanical damage to the crystals. Crystals at the very border of the bolus (within 200 μm of it) were slightly bleached in colour. This is likely due to LCP dehydration by means of the trapped air at the periphery of the sample. The appearance of the loaded disks changed slowly over the course of several hours, probably for the same reason. A 10 μl drop of bacteriorhodopsin-containing LCP was loaded in

a similar fashion, with a force of about 10 N for being applied several times in succession for several seconds each. The compressed sample thickness was measured to be only 20-30 μm. In this case the sample volume was sufficiently small to avoid overfilling the press plates. With 75 μm spatial spacing between shots, a 10 μl drop spread to 25 μm thickness (again a 23 mm diameter sample disk) would accommodate about 70,000 XFEL shots.

Figure S1 Silicon crystallography chip (Owen *et al.*, 2017) before (a) and after (b) being probed with the unattenuated SACLA XFEL beam ("top-hat" focal spot of 1.5 μm diameter, 480-500 μJ/pulse at the sample position, 10 fs pulses). Attenuating the X-ray beam to 18.6 % transmission considerably reduced the damage relative to that shown in (b), but did not eliminate it. The chip shown in the top image had previously been exposed at SACLA, but using an attenuated beam (13 % transmission) at 10 keV photon energy. The X-ray attenuation length of silicon is 134 μm at 10 keV but only 53 μm at 7.3 keV. The orange scale bar corresponds to 20 μm (a, b) the blue scale bar to 33 μm (b, marking the hole size).

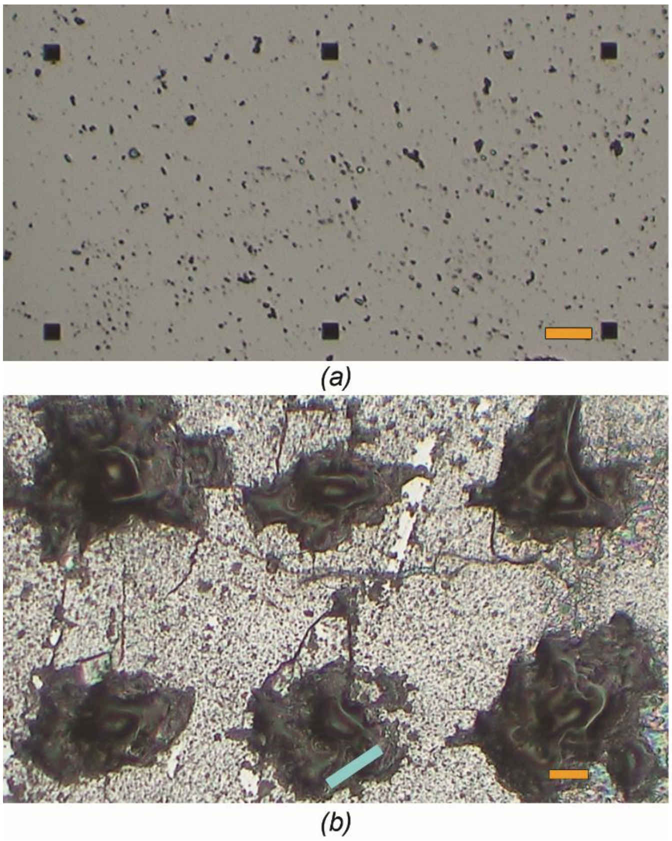

Figure S2 Cyclotene chip loaded with haemoglobin, after exposure to the SACLA XFEL beam of the same characteristics as in Figure S1. A silicon framework supports six individual windows of 5 μm thick glassy Cyclotene. An array of micron-sided holes in each window allows through-blotting of sample liquid, leaving only a very thin layer of crystals in solution on the window. An integral reservoir for solvent prevents desiccation for many hours. Windows were exposed to the XFEL beam in the order A, B, ..., F, with the resultant diffraction quality also deteriorating in that order.

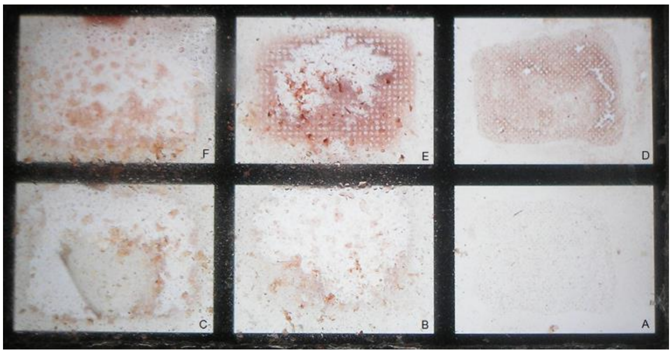

Figure S3 Top: Enlarged view of a section of Window A of Figure S2. The interleaved square holes of 20 and 10 µm are the blotting holes through the Cyclotene layer, spaced 75 µm on centres. The XFEL burns 5 to 15 µm diameter holes through the film. Bottom: From the holes, cracks extend radially outwards. The orange scale bar corresponds to 50 µm (a,b).

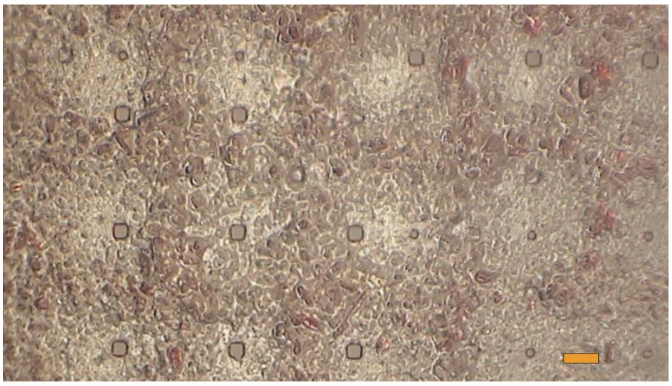

(a)

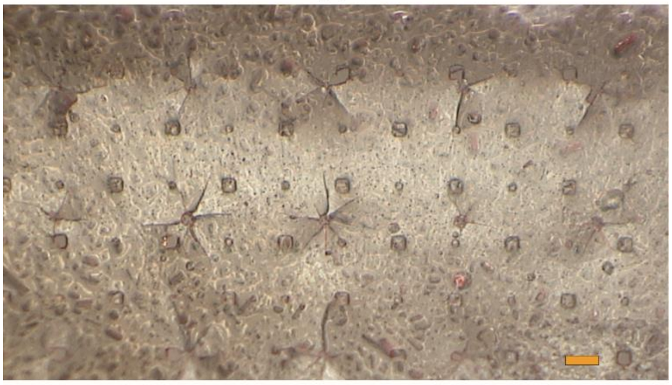

(b)

Figure S4 Frame real-time monitoring of the SACLA raster scan, exhibiting circular desiccation zones around holes burned by the XFEL beam through the SOS films. The orange scale bar corresponds to 200 µm.

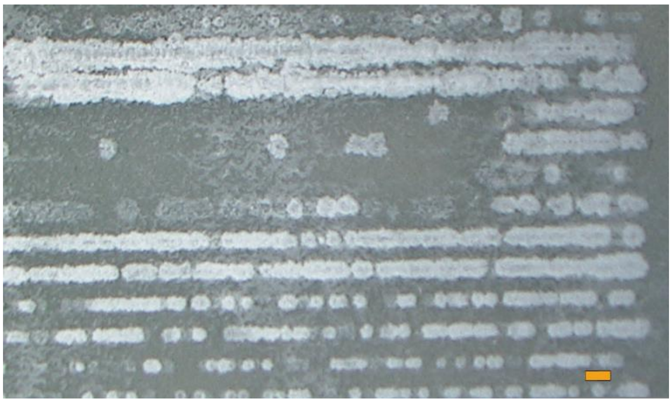

Figure S5 SOS mounting of membrane protein microcrystals grown in LCP. A few microliters of the viscous paste are deposited on the lower SOS sheet (a), which is then covered with the upper sheet and pressed between optically flat plates, as shown in (b) with the thrust plate of the press still raised above the base plate. A 10 µl volume of bacteriorhodopsin (bR) microcrystals grown in LCP (see Kovacsova *et al.*, 2017 for sample preparation) was spread to a thickness of 20-30 µm. (c). The fragile bR microcrystals are not damaged by this process (d). The jig for holding the SOS frames is that of Figure S7. The SOS frame is 30 mm square, inside clearance dimension.

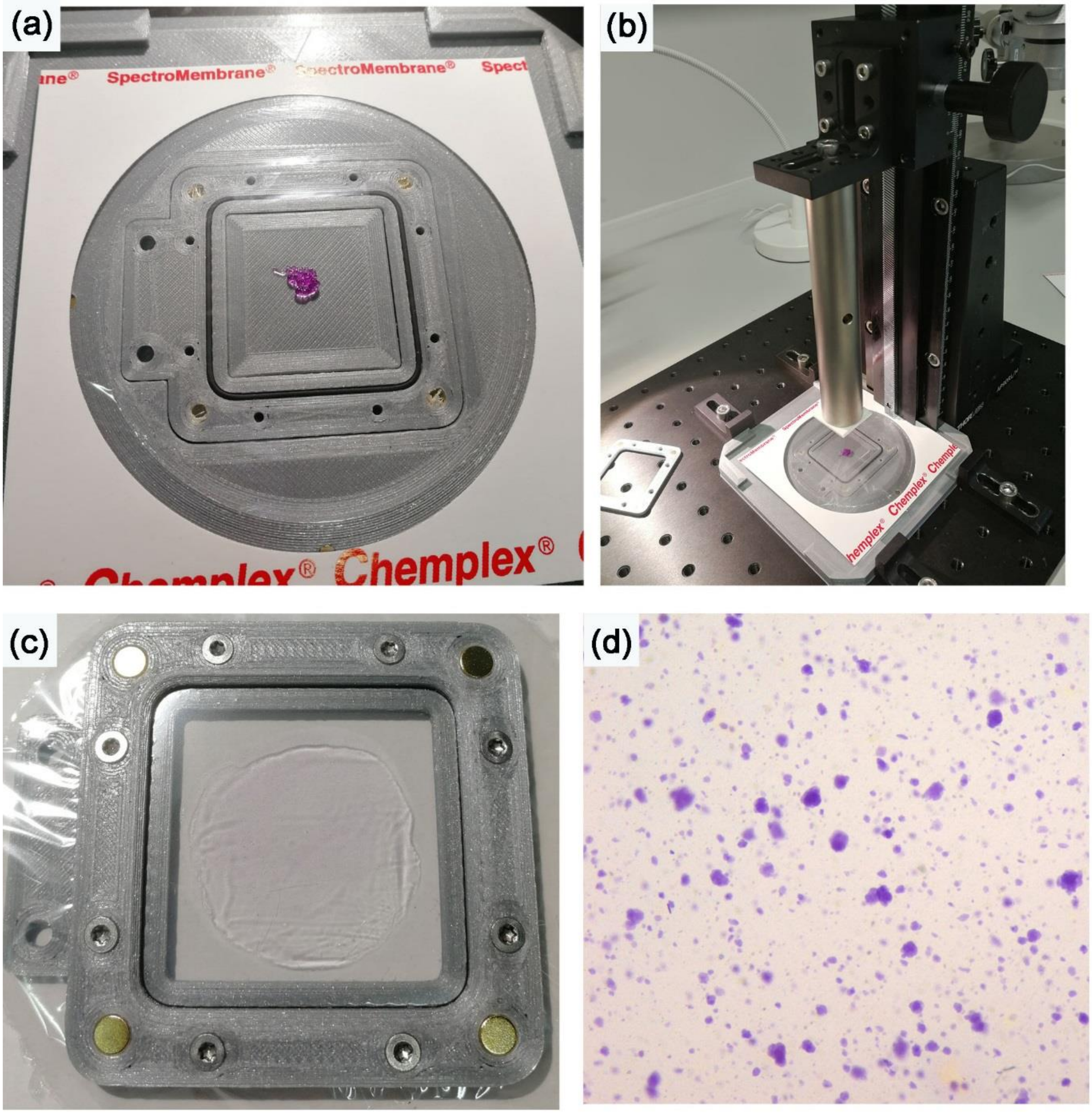

Figure S6 Mount for SOS sheets, shown assembled (left) and as individual components in the exploded view (right). The eight small disk magnets press permanently into the frames as does the O-ring into its groove in the back frame. The magnets pull the assembly snuggly together during the loading and assembly. Screws are then inserted to firmly clamp the two sheets together against the O-ring to hermetically seal the sample layer within the sheets. The structure is design to leave the assembled sheets under slight tension as the O-ring compresses, pulling them taut and planar.

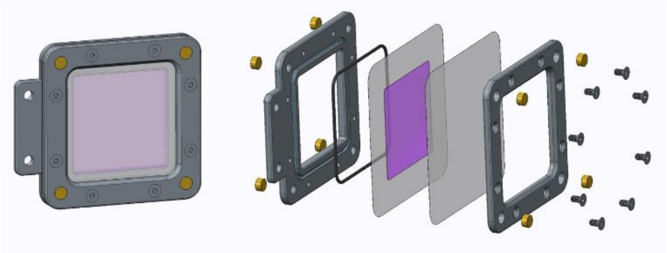

Figure S7 Exploded view depiction of the loading procedure (see text). The films shown are encased in tear-away paper frames for ease of handling, but this is not essential.

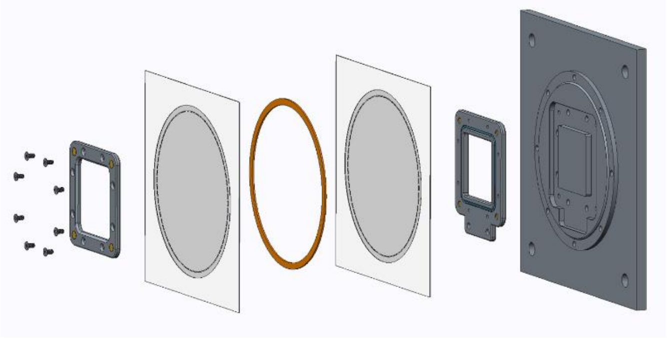

References

Owen, R. L., Axford, D., Sherrell, D. A., Kuo, A., Ernst, O. P., Schulz, E. C., Miller, R. J. & Mueller-Werkmeister, H. M. (2017). *Acta Cryst.* D**73**, 373-378.

Kovacsova, G., Grunbein, M. L., Kloos, M., Barends, T. R. M., Schlesinger, R., Heberle, J., Kabsch, W., Shoeman, R. L., Doak, R. B. & Schlichting, I. (2017). *IUCrJ* **4**, 400-410.
